# Integrated Traditional Chinese and Western medicine for ulcerative colitis with diabetes: A protocol for systematic review and meta-analysis

**DOI:** 10.1097/MD.0000000000024444

**Published:** 2021-01-29

**Authors:** Yu-Ze Lan, Ya-Li Bai, Xiang-Dong Zhu

**Affiliations:** Basic Medical College, Gansu University of Chinese Medicine, Lanzhou city, Gansu Province, China.

**Keywords:** diabetes, meta-analysis, traditional Chinese medicine, ulcerative colitis, Western medicine

## Abstract

**Background:**

This study aimed to access the efficacy and safety of integrated Traditional Chinese and Western medicine treatment for patients with ulcerative colitis (UC) combined diabetes.

**Methods:**

This protocol adheres to the preferred reporting items for systematic reviews and meta-analysis protocol statement. We plan to search 8 electronic databases to identify qualifying studies published from database inception until December 1, 2020. The software of EndNote reference manager (X9) will be used to study selection. A pre-developed standardized data collection form will be used to extract from all eligible studies. For included studies, the quality will be assessed by Cochrane Risk of bias tool. The RevMan 5.3 software (Copenhagen: The Nordic Cochrane Centre, The Cochrane Collaboration, 2014) developed by the Cochrane Collaboration will be used for all statistical analysis. If possible, meta-analysis will be undertaken for each of the outcomes. For continuous variable data, we will used mean differences with 95% confidence intervals (CIs) as summary statistics. For dichotomous variable data, we will calculate Mantel-Haenszel odds ratio with 95% CIs as summary statistics from the numbers of events in control and intervention groups. We will consider a result to be statistically significant if *P* < .05. If outcomes cannot be meta-analyzed, we will performer a descriptive analysis.

**Results:**

This study will be performed to test the efficacy and safety of integrated Traditional Chinese and Western medicine treatment for patients with UC combined diabetes.

**Conclusion:**

The results of our study will be published in a peer-reviewed journals, and we will promotion results in domestic and foreign conferences.

**Registration number:**

INPLASY2020120087

**Ethics and dissemination:**

As a systematic review and meta-analysis which based on previously published literature, ethical approval, and informed consent from patients are not required.

## 1 Introduction

As an immune-mediated chronic inflammatory bowel condition, ulcerative colitis (UC) is characterized by continuous, superficial inflammation of the colon.^[1,2]^ The etiology of the UC has not yet been determined and is generally believed to be influenced by environmental, genetic, and microbial factors.^[3]^ In worldwide, both the incidence and prevalence are increasing with a high economic burden of UC.^[4]^ The incidence of UC is 0.29% and the total annual cost is between $8.1 and 14.9$ billion in United States.^[5,6]^ The European has the highest incidence of UC in the word, such as Norway having a 0.51% incidence and the cost of UC ranges between €12.5 and €29.1 billion.^[5,6]^ The summary incidence rate of UC is 1.18 (95% confidence intervals (CIs): 0.81–1.56) per 100,000 person years in China that results from a systematic review (SR) and meta-analysis (MA).^[7]^ In general, UC brings a great burden to society and families.

In 2016, the International Diabetes Federation (IDF) estimates that the prevalence of diabetes will rise from 425 million people worldwide in 2017, to 629 million by 2045.^[8]^ But, the global diabetes data released by IDF in 2019 shows that there are already 463 million patients. This is likely to rise exponentially given the increasing prevalence of the condition. Diabetes and its associated complications consuming significant healthcare resources. Like UC, the etiology of diabetes is unclear and is often considered to be immune and genetic.

Current research has shown that the comorbid disease of UC included several immune mediated diseases, such as lupus, rheumatoid arthritis, psoriasis, and diabetes.^[9–12]^ And diabetes has the most frequent condition with UC. It is clearly that diabetes association with UC has epidemiological, pathogenetic, clinical, and therapeutic implications.^[13]^ Diabetes is significantly associated with UC both in children and in adult patients.^[14]^

Treatment of patient with UC depends mainly on the activity and location of disease.^[15]^ The first-line treatment of severe UC is corticosteroids with administered parenterally.^[16]^ The second-line treatments of severe UC are cyclosporine, tacrolimus, infliximab, or colectomy.^[17]^ Steroid treatment of UC is likely to induce high of hyperglycemia, especially in diabetic patients. Dehydration, electrolyte imbalance, sepsis, and parenteral nutrition caused by UC are important risk factors for hyperosmolar hyperglycemia status and diabetic ketoacidosis, the main complications of diabetes. Both conditions with high mortality rates are particularly dangerous clinically. Therefore, the Western medicine treatment of patients with UC complicated with diabetes is a great challenge. The integrated Traditional Chinese and Western medicine for UC with diabetes is likely to solve this problem. Some clinical research shows that the curative effect of integrated traditional Chinese and Western medicine is obviously better than that of simple Western medicine treatment, and didn’t increase the occurrence of adverse events.^[18,19]^ However, there is no high-level evidence about the efficacy and safety of integrated Chinese and Western medicine for UC complicated with diabetes. In this study, we will qualitatively and quantitatively examine the integrated Chinese and Western medicine treatment for patients with UC complicated with diabetes.

## 2 Methods

We drafted this protocol according to the Preferred Reporting Items for Systematic Reviews and Meta-Analysis Protocols (PRISMA-P) checklist.^[20]^ This SR started in December 2020 and was intended to be finished by June 2021. This study protocol has been funded on the INPLASY website (https://inplasy.com/inplasy-2020-12-0087/). If this protocol needs to revise, we will also make corrections simultaneously in INPLASY protocol. In addition, the proposed review will be conducted in accordance with the “Preferred Reporting Items for Systematic Reviews and Meta-Analyses (PRISMA).”^[21]^

### 2.1 Search strategy

We plan to search 8 electronic databases (4 English databases: PubMed, Embase, Cochrane Library, and Web of Science; 4 Chinese databases: China biology Medicine [CBM], China National Knowledge Infrastructure [CNKI], Wanfang Data and Chinese Scientific Journal Database [VIP]) to identify qualifying studies published from database inception until December 1, 2020. The search strategy will be formulated by library search specialist and epidemiologist. The Medical Subject Headings (MeSH) and free words will be used to search in the above databases. Boolean Logic (AND, OR) will be combined using in the search terms when needed. Human studies and peer-reviewed journal articles published in English or Chinese will be restricted in our search. The search strategies for PubMed can be found in Table 1. In addition, we will manually search the reference lists of all included studies for any further potentially relevant studies.

**Table 1:** Search strategy of PubMed.

Search	Query
#1	“Colitis, Ulcerative”[Mesh]
#2	“Ulcerative Colitis”[Title/Abstract] OR “Ulcerative Colitiz”[Title/Abstract] OR “Ulcerative Colitises”[Title/Abstract] OR “Ulcerative Colitizes”[Title/Abstract] OR “Idiopathic Proctocolitis”[Title/Abstract] OR “Idiopathic Proctocolitises”[Title/Abstract] OR “Colitis Gravis”[Title/Abstract] OR “Colitis Gravises”[Title/Abstract]
#3	#1 OR #2
#4	“Diabetes Mellitus”[Mesh] OR “Diabetes Mellitus, Type 2”[Mesh] OR “Diabetes Mellitus, Type 1”[Mesh] OR “Diabetes Mellitus, Lipoatrophic”[Mesh]
#5	diabetes [Title/Abstract]
#6	#4 OR #5
#7	#3 AND #6
#8	“Medicine, Chinese Traditional”[Mesh]
#9	“traditional Chinese medicine”[Title/Abstract] OR “TCM”[Title/Abstract] OR “Integrated traditional Chinese and Western medicine”[Title/Abstract] OR “Medicine, Chinese Traditional”[Title/Abstract] OR “Traditional Medicine, Chinese”[Title/Abstract] OR “Zhong Yi Xue”[Title/Abstract]
#10	#8 OR #9
#11	#7 AND #10

### 2.2 Study selection

**Figure 1:**
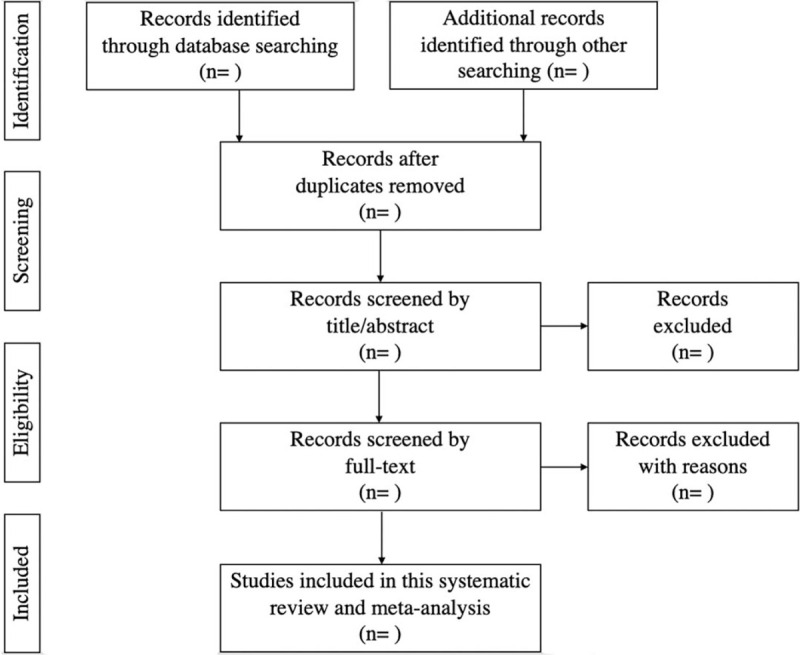
Flow chart of study selection.

The software of EndNote reference manager (X9) will be used to study selection. Firstly, retrieved records from databases searches will be entered into this software. Secondly, duplications will be removed through machines and manual. Thirdly, we will screen all titles and abstracts for potentially relevant studies. Fourth, we will be retrieved and screened for relevant studies by the full-texts. The reasons for exclusion of any articles will be noted. All of the screen process will be performed independently by 2 authors. The third author will be consulted if consensus on eligibility cannot be achieved. A PRISMA flow diagram presented the details of the selection process (Fig. 1).

### 2.3 Inclusion criteria

#### 2.3.1 Type of studies

Randomized controlled trials (RCTs) will be included in this review. Self-controlled studies, non-RCTs, randomized crossover studies, quasi-randomized trials, case report, case series, and cross-sectional studies will be excluded.

#### 2.3.2 Type of participants

All participants are diagnosed with UC by medical history, endoscopic parameters, clinical evaluation, and histopathology.^[22]^ And infectious colitis will be excluded careful. In addition, patients with UC also suffer from diabetes (included type 1 diabetes and type 2 diabetes). The diagnostic criteria for diabetes are typical symptoms of diabetes (polydipsia, polyuria, polyphagia, and weight loss) plus random plasma glucose ≥11.1 mmol/L or fasting plasma glucose (FPG) ≥7.0 mmol/L or oral glucose tolerance test (OGTT) 2-Hour plasma glucose (2hPG) ≥11.1 mmol/L.^[23]^ All patients must be adults (≥18 years). There will be regardless about sex, region, education, economic, and another factor.

#### 2.3.3 Type of interventions

The control group received conventional treatment of Western medicine, and did not restrict the type of drugs, dosage form, frequency, and course of treatment. The intervention measures of the experimental group were combined with Traditional Chinese Medicine (TCM) on the basis of the control group, included Chinese herbal medicine and Chinese patent medicine. Other intervention measures of TCM will be ruled out, such as acupuncture, moxibustion, massage.

#### 2.3.4 Type of outcomes

##### 2.3.4.1 Primary outcomes

Total effective rate (referring to the diagnostic criteria of ulcerative colitis in the Consensus Opinions on Diagnosis and Treatment Standards of Inflammatory Bowel Disease in China).^[24]^ (Total effective rate = number of complete remission + effective number)/total number × 100%. Complete remission is defined as the clinical symptoms are disappearance, and the mucosa is found to be roughly normal by colonoscopy reexamination. Effective represents the basic disappearance of clinical symptoms, mild inflammation of mucosa, or pseudopolyp formation by colonoscopy reexamination. Ineffective represents no improvement of clinical symptoms, endoscopy, and pathological examination results after treatment.

##### 2.3.4.2 Secondary outcomes

Recurrence rate, symptom score, colonoscopic score, serum bloody test (such as antineutrophil cytoplasmic antibodies, ANCA), mucosal healing rate, and incidence of adverse reactions.^[25–28]^

### 2.4 Data extraction

A pre-developed standardized data collection form will be used to extract from all eligible studies. The relevant information including title, first author, publication year, sample size, average age, sex ratio, average course of disease, drug type, dosage form, frequency, course of treatment, and outcome indicators. In addition, important citations, funding agencies and potential conflicts of interest will also be collected. We will contact the corresponding author by email if the required data are missing or unclear. Data will be independently extracted by 2 reviewers. There will be discussed between 2 reviewers if discrepancies arise. If necessary, a third reviewer will be consulted to achieve consensus.

### 2.5 Quality appraisal of included studies

The quality of all included studies will be independently assessed by 2 reviewers, and any disagreements will be determined by the third author. For included studies, the quality will be assessed by Cochrane Risk of bias tool.^[29]^ This tool consists of the following 7 questions: random sequence generation (selection bias), allocation concealment (selection bias), blinding of participants, blinding of outcome assessment (performance bias), incomplete outcome data (attrition bias), selective reporting (reporting bias), and other bias (“other bias” is determined by consensus of the investigators). Each question has 3 answers: “Yes” (low risk of bias), “No” (high risk of bias), or “Unclear” (lack of information or uncertainty over the potential bias).

### 2.6 Data synthesis and assessment of heterogeneity

If possible, MA will be undertaken for each of the outcomes. For continuous variable data, we will use mean differences with 95% CIs as summary statistics. If studies had used different measurement instruments or units to measure an outcome, we plan to use the standardized mean difference. For dichotomous variable data, we will calculate Mantel-Haenszel odds ratio with 95% CIs as summary statistics from the numbers of events in control and intervention groups. We will consider a result to be statistically significant if *P* < .05. If outcomes cannot be meta-analyzed, we will performer a descriptive analysis.

We will explore clinical and statistical sources of heterogeneity among the different groups of RCTs by *I*^2^ statistics and chi-squared test (<25% deemed low, 25–50% deemed moderate, and >50% deemed high). If heterogeneity is moderate, we will use random effects model to analysis. If heterogeneity is high, we will perform a subgroup analysis. We also will performer a descriptive analysis if cannot perform subgroup analysis.

Where ≥10 RCTs are included in a MA, we will assess the publication bias by funnel plot.^[30]^ And Eggers and Beggs test also will be used to evaluation of potential publication bias.

### 2.7 Subgroup analysis

If necessary or possible, results will be analyzed for the following subgroups: type of diabetes: type 1 diabetes, type 2 diabetes; age groups: 18–45 years, 46–60 years, 61–75 years, >75 years; sex: man, woman.

The RevMan 5.3 software (Copenhagen: The Nordic Cochrane Centre, The Cochrane Collaboration, 2014) developed by the Cochrane Collaboration will be used for all statistical analysis.

### 2.8 Overall quality of evidences

The quality of evidence for outcomes will be assessed using the Grading of Recommendations Assessment, Development and Evaluation approach.^[31]^ The quality of evidence will be divided into four levels: “high,” “moderate,” “low,” and “very low.”

## 3 Discussion

TCM can adjust of qi movement, balances Yin and Yang of the body, and restores the normal physiological functions of the Zang-fu organs (viscera) through syndrome differentiation and treatment. Modern pharmacology also has proved that part of TCM has the function of immune regulation, anti-inflammation, promote metabolism, and improve microcirculation.^[32–34]^ It has been studied that integrated Traditional Chinese and Western medicine for patients with UC combined diabetes.^[18,19]^ This SR and MA can provide scientific evidence for clinical and future studies on therapy of integrated Traditional Chinese and Western medicine for patients with UC combined diabetes. We estimate that this SR and MA can be completed in June 2021. The results of this study will be published in peer-reviewed journals. And we will promotion results in domestic and foreign conferences.

## Author contributions

**Data curation:** Yu-Ze Lan.

**Formal analysis:** Yu-Ze Lan, Ya-Li Bai.

**Funding acquisition:** Xiang-Dong Zhu.

**Methodology:** Yu-Ze Lan, Ya-Li Bai.

**Project administration:** Yu-Ze Lan, Xiang-Dong Zhu.

**Resources:** Yu-Ze Lan, Ya-Li Bai.

**Software:** Yu-Ze Lan, Ya-Li Bai.

**Supervision:** Xiang-Dong Zhu.

**Validation:** Xiang-Dong Zhu.

**Writing-original draft:** Yu-Ze Lan.

**Writing-review & editing:** Xiang-Dong Zhu.

## Abbreviations

Abbreviations: CIs = confidence intervals, IDF = International Diabetes Federation, MA = meta-analysis, PRISMA = Preferred Reporting Items for Systematic Reviews and Meta-Analyses, RCTs = randomized controlled trials, SR = systematic review, TCM = Traditional Chinese Medicine, UC = ulcerative colitis.
